# Net Improvement of Correct Answers to Therapy Questions After PubMed Searches: Pre/Post Comparison

**DOI:** 10.2196/jmir.2572

**Published:** 2013-11-08

**Authors:** Kathleen Ann McKibbon, Cynthia Lokker, Arun Keepanasseril, Nancy L Wilczynski, R Brian Haynes

**Affiliations:** ^1^McMaster UniversityDepartment of Clinical Epidemiology and BiostatisticsHealth Information Research UnitHamilton, ONCanada

**Keywords:** information services, information storage and retrieval, Internet, Medline, physicians, primary health care

## Abstract

**Background:**

Clinicians search PubMed for answers to clinical questions although it is time consuming and not always successful.

**Objective:**

To determine if PubMed used with its Clinical Queries feature to filter results based on study quality would improve search success (more correct answers to clinical questions related to therapy).

**Methods:**

We invited 528 primary care physicians to participate, 143 (27.1%) consented, and 111 (21.0% of the total and 77.6% of those who consented) completed the study. Participants answered 14 yes/no therapy questions and were given 4 of these (2 originally answered correctly and 2 originally answered incorrectly) to search using either the PubMed main screen or PubMed Clinical Queries narrow therapy filter via a purpose-built system with identical search screens. Participants also picked 3 of the first 20 retrieved citations that best addressed each question. They were then asked to re-answer the original 14 questions.

**Results:**

We found no statistically significant differences in the rates of correct or incorrect answers using the PubMed main screen or PubMed Clinical Queries. The rate of correct answers increased from 50.0% to 61.4% (95% CI 55.0%-67.8%) for the PubMed main screen searches and from 50.0% to 59.1% (95% CI 52.6%-65.6%) for Clinical Queries searches. These net absolute increases of 11.4% and 9.1%, respectively, included previously correct answers changing to incorrect at a rate of 9.5% (95% CI 5.6%-13.4%) for PubMed main screen searches and 9.1% (95% CI 5.3%-12.9%) for Clinical Queries searches, combined with increases in the rate of being correct of 20.5% (95% CI 15.2%-25.8%) for PubMed main screen searches and 17.7% (95% CI 12.7%-22.7%) for Clinical Queries searches.

**Conclusions:**

PubMed can assist clinicians answering clinical questions with an approximately 10% absolute rate of improvement in correct answers. This small increase includes more correct answers partially offset by a decrease in previously correct answers.

## Introduction

Medline indexed 760,903 new articles in 2012, bringing their total to just under 20 million articles. The number of journals indexed by Medline has grown by 50% in the past 20 years [1]. During 2012, 2.2 billion Medline searches were done. Although quantification of this information overload in the health care literature is limited [2], it is widely perceived as an obstacle for physicians practicing evidence-based medicine and searching for answers to their clinical questions [3].

The 6S pyramid of evidence from health care research describes a range of tools and resources to assist physicians in accessing or retrieving relevant research evidence. The pyramid is structured so that original studies form the base and are topped by, in ascending order of clinical usefulness, synopses of studies, syntheses (systematic reviews), synopses of syntheses, summaries (evidence-driven online texts), and systems (eg, clinical decision support systems) [4]. In addition to published evidence, colleagues and textbooks are often first-line information resources used by physicians [5-7] because these give answers most efficiently [7]. Although higher levels of evidence (eg, meta-analyses or clinical summaries) are more clinically useful, this kind of information is not available for many clinical questions and physicians often need to search the primary literature [8]. Physicians report substantial use of PubMed or Medline through other vendors. Davies [9] reported that 81% of US physicians in 2007, 77% of UK physicians, and 76% of Canadian physicians used PubMed or Medline occasionally or often to support their practices.

Research has shown that published original studies and reviews can provide clinicians with answers to their clinical questions [10-13] and lead to changes in patient care [13-15]. Medline searches helped medical and nurse practitioner students answer simulated clinical questions [12]. A virtual library containing Medline, textbooks, and clinical guidelines helped physicians find relevant information on clinical questions [10]. A study of 33 emergency department residents, however, found that Google search results gave participants a false sense of security, resulting in a dramatic increase in confidence in their answers. Google searches translated into more correct responses to simulated questions, but also slightly more wrong answers after searching [16].

Other studies have reported negative effects of information searching on physician responses to clinical questions. McKibbon and Fridsma [17] found that 11% of answers to clinical questions went from correct before searching to incorrect after searching when clinicians used their preferred online resources. Hersh and colleagues [12,18] found rates of correct-to-incorrect answers of 4.5% and 10.5% using Medline in 2 studies.

Search filters have been developed to help clinicians search the primary literature. These filters are rigorously developed and validated to increase the yield of clinically relevant articles based on research methods or clinical content. The Health Information Research Unit at McMaster University has developed filters for detecting primary studies for therapy [19,20], diagnosis [21,22], economics [23], prognosis [24,25], etiology [26,27], systematic reviews [28], and studies in mental health [29]. A number of filters have been made available on PubMed in the Clinical Queries interface [30] and the Special Queries feature [31]. A recent study comparing search retrieval from the main PubMed screen and from Clinical Queries found that Clinical Queries returned fewer studies, more of which were methodologically sound [32].

The objective of this pragmatic study was to determine if differences exist in the rate of correct answers to clinical questions when primary care physicians use the PubMed main screen or the Clinical Queries feature of PubMed for searches. Specifically, do searches done by primary care physicians through the PubMed main screen or through Clinical Queries give different rates of correctness of answers to clinical questions related to therapy?

## Methods

### Standardized Questions

To assess if PubMed provided correct answers to clinical questions related to therapies, standard clinical questions with answers based on recent systematic reviews were developed. The reviews were selected from a database of clinical research from 125 journals preappraised for methodological rigor [33] and rated by a worldwide panel of practicing clinicians for relevance to clinical practice and newsworthiness. Reviews relevant to general practice from the first 6 months of 2011, with clinical relevance and newsworthiness ratings >5 of 7, were assessed to determine whether they reported a definitive answer to the clinical question at hand.

In all, 24 standard questions were devised and iteratively tested on 3 physicians. The physicians were 2 experienced general practitioners and 1 experienced general internist. A fourth general internist also reviewed the questions. The physicians provided input on clinical applicability, perceived difficulty of the question, and relevance to practice for each question. Revised questions were then piloted on 2 general practitioners who provided further feedback. Questions were dropped if they were perceived by the clinicians as being too difficult or easy to answer, not relevant to general practice, or if the answer was perceived to be controversial. The remaining 14 questions are presented in Table 1.

**Table 1:** Standardized questions provided to general practitioners based on systematic reviews published in early 2011.

Question	Evidence-based answer
1. In adults wishing to quit smoking, is varenicline (Champix) better than bupropion in terms of successful smoking cessation? [34]	Yes
2. Should antidepressants be prescribed for patients >18 years who are diagnosed with minor/subthreshold depression according to standardized criteria? [35]	No
3. In a middle-aged patient who is at high risk for cardiovascular events, does clopidogrel plus aspirin provide safer and more effective protection from cardiovascular events than aspirin alone? [36]	No
4. Over the long term, can daily low-dose aspirin reduce mortality caused by a range of cancers? [37]	Yes
5. Does estrogen therapy increase the risk of kidney stones in otherwise healthy postmenopausal women (>60 years)? [38]	Yes
6. Can maternal depression during pregnancy lead to preterm birth and low birth weight? [39]	Yes
7. Is it safe and effective to progressively increase statin therapy intensity to lower LDL^a^ levels and reduce the risk of occlusive vascular events in patients with high LDL levels? [40]	Yes
8. Does dietary supplementation with folic acid to lower homocysteine levels prevent cardiovascular events in high-risk adults? [41]	No
9. For a patient at high risk of cardiovascular events and who is concerned about erectile dysfunction, can you prescribe ACE^b^-inhibitors, angiotensin receptor blockers, or calcium channel blockers without worrying about his sexual functioning? [42]	Yes
10. Compared to other antihypertensive drugs, is hydrochlorothiazide 12.5 to 25 mg/day suitable as first-line drug therapy for the treatment of adult hypertension? [43]	No
11. For an adult patient with type 2 diabetes who needs thiazolidinedione treatment, is pioglitazone a safer treatment than rosiglitazone? [44]	Yes
12. Does treatment of periodontal disease (simple dental scaling and root planing) in pregnant women reduce their risk of preterm delivery? [45]	No
13. In patients with chronic back pain caused by disk degeneration, does spinal fusion surgery result in better long-term benefits than nonsurgical approaches? [46]	No
14. Should I advise patients with asthma to double their regular dose of inhaled corticosteroids as a first step in dealing with an exacerbation? [47]	No

^a^LDL: Low Density Lipoprotein

^b^ACE: angiotensin-converting enzyme

### Recruitment

Practicing physicians self-identified as general practitioners, family practitioners, or primary care general internal medicine practitioners who were registered with the McMaster Online Rating of Evidence (MORE) [48] system were emailed invitations to participate in the online research study. Invitations were sent to 528 physicians in November 2011 with up to 2 reminders sent by the end of January 2012. Participants were provided with certification of 1 hour of continuing medical education credit for completing the study.

### Survey

**Figure 1:**
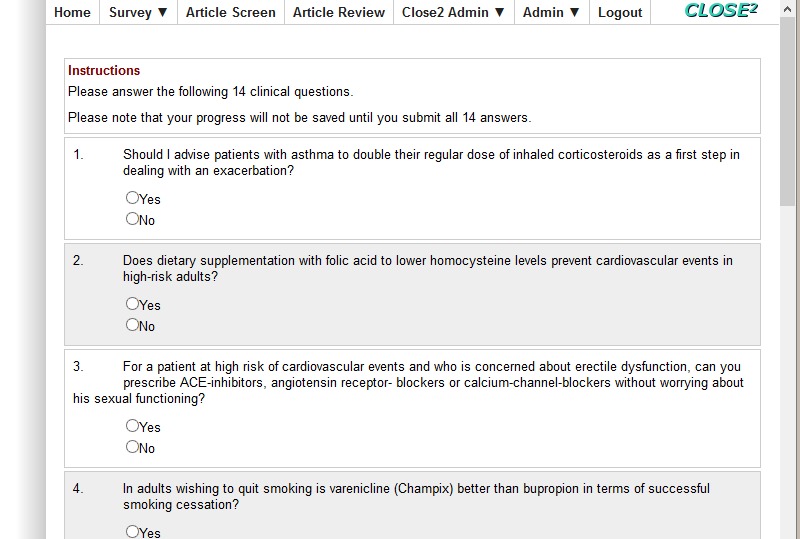
Entry screens asking for answers to 14 clinical questions. Each participant completed this task twice (before and after the search process).

**Figure 2:**
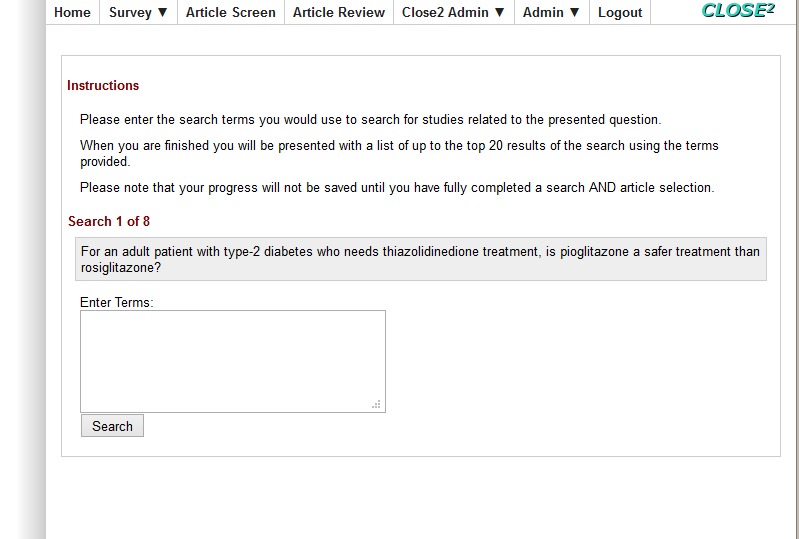
Term entry screen for both searching tasks.

Participants were sent an Internet link to the survey that required them to sign into our information production system of high-quality clinical articles using their system passwords, which started the task (Figure 1). After providing consent, physicians were asked to answer the 14 clinical questions with a yes or no answer (Table 1). They were then asked to search for information on 4 of the questions (Figure 2). The 4 questions included 2 that they had initially answered correctly and 2 that they had answered incorrectly; we did not indicate to the participant if his or her answers were correct. Three separate computer-generated randomizations were involved: (1) questions for searching were selected randomly, (2) the questions were sent to PubMed main screen or Clinical Queries randomly (1 correct and 1 incorrect in each), and (3) the order in which the clinicians searched was randomized. The 2 interfaces were conduits to the PubMed search system and all the search algorithms functioned in their usual manner; the entered terms were passed into PubMed with or without Clinical Queries filters.

Because our questions were treatment questions, we used the therapy category of the Research Methodology filter of Clinical Queries. We were interested in clinicians searching for answers to clinical questions; therefore, we used the narrow Clinical Queries. The narrow search filters are designed for clinical care because they retrieve a good proportion of potentially relevant citations while keeping the number of nonrelevant citations to a minimum (sensitivity of 93% and specificity of 97% [19]). The broad clinical filters are designed for researchers and meta-analysts who want to retrieve the highest proportion of relevant citations with less regard to retrieving nonrelevant citations.

For each question, participants were asked to enter search terms into a textbox. If participants were unhappy with the retrieval, they could alter their search terms and submit a new search.

After each of the 4 searches, the first 20 results were presented. The participant was blinded as to which PubMed interface the retrieval came from. They could view the abstract of the article in the same window by selecting the title of the article. Participants were asked to select the top 3 articles most important to forming/supporting their answer. After the 4 searches were performed and articles were selected, the participants were given the 14 questions again and asked to answer them with yes or no. The study was approved by the McMaster University Hamilton Health Sciences/Faculty of Health Sciences Research Ethics Board.

### Statistical Analysis

The primary outcome of the study was the difference in the proportion of correct answers before and after searching. Secondary outcomes were the proportion of questions searched that went from incorrect to correct and correct to incorrect, the proportion of questions without searches that went from correct to incorrect and incorrect to correct, and the time taken to complete the project tasks.

Based on previous studies, starting proportions of answers to clinical questions were 27% correct (n=557) [10] and 40% correct (n=46) [17]. These studies found a rate of answers going from correct to incorrect of 7% [18] and 11% [17], respectively.

We anticipated an approximately 10% change in correct to incorrect answers; therefore, we set a 5% absolute difference between search modes as clinically interesting. This gave us a sample size of 522 searches for the correct group and 459 for the incorrect group to detect a 5% difference in search modes with 80% power.

The Mantel-Haenszel test for matched pairs, stratified by participant and by question, was used to determine the odds ratio of changing a response by using Clinical Queries searches versus PubMed main screen searches. A posteriori we recognized that question 6 was a prognosis question rather than a treatment question. Given that the Clinical Queries searches used a therapy filter, we performed our analysis including this question and the sensitivity analysis without this question. In the entire dataset, only 1 of 29 participants (3%) presented with question 6 changed their answer (correct to incorrect with Clinical Queries).

### Study Quality

Articles selected by the participants as being relevant to answering their question were independently assessed in duplicate for methodological criteria outlined below. Disagreements were resolved through consensus.

A therapy study is methodologically sound if it meets these 3 criteria:

1. Random allocation of participants to comparison groups;
2. Outcome assessment of at least 80% of those entering the investigation accounted for in one major analysis at any given follow-up assessment; and
3. Analysis consistent with study design.

A systematic review of therapy studies is methodologically sound if it meets these 6 criteria:

1. Explicit statement of clinical topic;
2. Question refers to treatment;
3. Methods are described in report body (not just the abstract);
4. More than one major database searched or Cochrane CENTRAL searched;
5. Explicit inclusion/exclusion criteria; and
6. One or more articles meet criteria set out for therapy studies (listed previously).

## Results

### Summary

During recruitment, 528 physicians were invited to take part in the study; 143 (27.1%) provided consent, 110 of whom (21.0% of those invited and 77.6% of those who consented) completed the study tasks (24 abandoned the task after the first search and 9 did not perform any searches). Two participants (1.8%) answered all 14 questions correctly; consequently, they were directed to search for only 2 questions. At baseline, participants answered 62.3% (95% CI 59.8%-64.7%) of the questions correctly.

Time to complete the tasks was calculated based on the time the participant signed in to the website until the time they submitted the survey. If the participant logged off without clicking the submit button, the timer continued to count. As such, 16 observations were more than 100 minutes, ranging from 119 to 103,786 minutes (72 days). We selected a cutoff of 100 minutes as a likely point at which the tasks were not completed in 1 sitting. The remaining 95 participants completed the tasks within 6 to 76 minutes (mean 24.5 minutes, 95% CI 21.4-27.5).

### Searches

During the study, 440 searches were executed, 222 (50.5%) answered correctly and 218 (49.5%) answered incorrectly initially. For questions selected for searching, baseline responses were 50.0% correct in both groups by design. After searching, responses were correct for 61.4% (95% CI 55.0%-67.8%) of questions for the PubMed main screen group, and 59.1% (95% CI 52.6%-65.6%) for the Clinical Queries group. We found no differences in the rate of answers going from incorrect to correct for the PubMed main screen searches (45/220, 20.5%) compared with the Clinical Queries searches (39/220, 17.7%) (Table 2). Both sets of searches also had an approximate 9% rate of going from correct to incorrect: 21 of 220 (9.5%) for PubMed main screen and 20 of 220 (9.1%) for Clinical Queries (Table 2). Searches resulted in a net gain of 11.4% (95% CI 2.1%-20.4%) in correct answers for PubMed main screen searches and 9.1% (95% CI –0.2% to 18.2%) for Clinical Queries searches.

The odds of changing an answer with a Clinical Queries search versus a PubMed main screen search was not different for questions that were initially correct or initially wrong stratified by user or by question (*P*>.05) (Table 3). Sensitivity analysis removing question 6 (a prognosis question) did not alter the results.

**Table 2:** Proportion of the PubMed main screen search group and PubMed Clinical Queries search group that changed answers (correct to incorrect or incorrect to correct) or kept them the same (correct or incorrect).

Search platform	Answers stayed the same				Answers changed			
	Correct		Incorrect		Correct to incorrect		Incorrect to correct	
	Searches, n	% (95% CI)	Searches, n	% (95% CI)	Searches, n	% (95% CI)	Searches, n	% (95% CI)
PubMed main screen (n=220)	90	40.9 (34.4-47.4)	64	29.1 (23.1-35.1)	21	9.5 (5.6-13.4)	45	20.5 (15.2-25.8)
PubMed Clinical Queries (n=220)	91	41.4 (34.9-47.9)	70	31.8 (25.7-38.0)	20	9.1 (5.3-12.9)	39	17.7 (12.7-22.7)

**Table 3:** Mantel-Haenszel odds ratios for changed answers based on searches through Clinical Queries vs the PubMed main screen.

Starting answer		n	OR (95% CI)
**Correct**			
	Participant	33	0.94 (0.48-1.86)
	Question	13	1.14 (0.55-2.35)
**Incorrect**			
	Participant	52	0.79 (0.46-1.37)
	Question	13	0.80 (0.47-1.36)

### Nonsearched Questions

For questions answered before and after searching but without intervening searches, an average of 65.4% (95% CI 62.8%-68.0%) were correct at baseline, 64.6% (95% CI 62.0%-67.2%) were correct at the end of the study across the 14 questions, 4.0% (95% CI 2.3%-5.6%) went from correct to incorrect, and 3.1% (95% CI 2.2%-4.1%) went from incorrect to correct. There was variability in baseline performance across questions (Table 4). Without searches, the odds of changing an answer from correct to incorrect was lower (OR 0.06, 95% CI 0.05-0.08) than changing from incorrect to correct (OR 0.11, 95% CI 0.08-0.13; *P*=.002).

**Table 4:** Responses for questions without searches.

Question	Correct to correct, % (n)	Incorrect to incorrect, % (n)	Correct to incorrect, % (n)	Incorrect to correct, % (n)	Responses, n
1	64 (55)	28 (24)	5 (4)	3 (3)	86
2	83 (70)	8 (7)	1 (1)	7 (6)	84
3	74 (59)	23 (18)	3 (2)	1 (1)	80
4	68 (50)	27 (20)	3 (2)	3 (2)	74
5	25 (16)	66 (43)	5 (3)	5 (3)	65
6	78 (64)	16 (13)	5 (4)	1 (1)	82
7	77 (64)	17 (14)	4 (3)	2 (2)	83
8	82 (75)	10 (9)	4 (4)	3 (3)	91
9	45 (38)	45 (38)	5 (4)	5 (4)	84
10	18 (12)	79 (53)	1 (1)	1 (1)	67
11	64 (53)	31 (26)	2 (2)	2 (2)	83
12	45 (35)	38 (29)	13 (10)	4 (3)	77
13	93 (78)	4 (3)	2 (2)	1 (1)	84
14	45 (33)	49 (36)	3 (2)	4 (3)	74

### Study Quality

Clinical Queries were developed to retrieve clinically useful studies based on study design. Therapy filters retrieve citations based on the article being a randomized controlled trial. Therefore, we were interested in determining if the participants identified studies with strong methods (ie, randomized controlled trials or reviews that analyzed randomized controlled trials) when presented with the first 20 retrievals. The participants were asked to identify the 3 most important articles that provided evidence to answer the clinical question they were addressing. Overall, the PubMed main page group tagged 334 articles as important and the Clinical Queries group tagged 321 articles. Table 5 shows the number of treatment articles and systematic review articles tagged as important to the questions asked. Articles selected from the PubMed main screen searches and the Clinical Queries searches did not differ in the number of review treatment articles selected as important or the number of original or review articles that had strong methods.

**Table 5:** Number of articles with strong methods (randomized controlled trials or systematic reviews of randomized controlled trials) identified as important by the clinician searchers.

Methodologic rigor for articles on treatment identified as influencing decisions	PubMed main screen	Clinical Queries
Proportion of original articles meeting criteria (strong methods for therapy)	45/100 (45.0%)	58/118 (49.1%)
Proportion of review articles meeting criteria (strong methods for therapy)	42/124 (33.8%)	42/124 (33.8%)

## Discussion

Although we sought to show that searches with PubMed Clinical Queries were associated with more correct answers to clinical questions than were PubMed main screen searches, we did not find any differences. This may be because we did not meet our sample size of approximately 1000 searches as originally calculated. Another explanation for these results may be the training and experience of the study participants. All were practicing clinicians and were registered with the MORE system, wherein they evaluate and rate clinician-ready health research studies. Also, this study was done on the Internet. The participants likely had strong computer and Internet skills and were probably skilled users of PubMed and the clinical research literature. Therefore, the study participants may be the least likely group of clinicians who could benefit from using Clinical Queries. Naïve users or new clinicians, such as interns and residents, or those clinicians less skilled at the assessment and application of research findings may derive more benefit from the Clinical Queries searches. Time is also a major factor in seeking answers to clinical questions. If the time to seek answers had been more tightly constrained in the study (we did not have time limits on the tasks) we may have found a larger difference between the correctness of the answers found with standalone PubMed searches and searches using Clinical Queries.

However, this study does show that PubMed, either on its own or using Clinical Queries, helps clinicians answer clinical questions with increased accuracy. For questions that clinicians answered twice without searching, the rate of correct answers stayed the same (65.4% correct at first answer and 64.6% correct on second answer). With searching, clinicians improved their rate of correct replies. Their answers went from 50.0% correct (set by the study) to 60.2% correct (59.1% for PubMed Clinical Queries searches and 61.4% for PubMed main screen, *P*=.60).

Our findings are consistent with other studies that found use of information resources is associated with an increase in accuracy of clinical answers [10,12,17,18]. This increase is often in the range of an absolute 10% improvement. However, the increase in the number of correct answers with searching is almost always a combination of approximately 20% of answers going from an initial incorrect answer to correct at the same time as a 10% rate going from an initial correct answer to an answer that is incorrect.

We also found some change in answers for questions that were not the basis of searches in this study. The steady state of the study participants being correct approximately 65% of the time was almost balanced with 4% of the questions going from correct to incorrect and 3% going from incorrect to correct. This phenomenon of changing answers should be taken into account for studies that are based on outcomes of correct answers to clinical questions.

We have shown that complex searching studies with multiple tasks can be done through the Internet and we were able to recruit clinicians for searching trials. Our participants spent an average of 25 minutes online. During this time, they answered 14 yes/no questions twice, completed 4 PubMed searches, and selected articles of importance to clinical questions. Our methods were strengthened in that we blinded participants to the purpose of the study, kept the clinicians blinded to their initial answers and whether they were using Clinical Queries or not, and performed blinded and duplicate readings in the assessment of the methodological strength of the original and review articles on treatment. We also randomized 3 procedures (choice of question to be searched, order of using PubMed main screen or Clinical Queries searches, and questions that were sent to the 2 searching methods).

The questions we used in the study were based on strong evidence from current systematic reviews, and they were pretested with various physician groups. However, despite these strengths, our questions were not questions that arose in the participants’ daily practices.

Future research needs to be done to improve the quality of search tools and their ability to maximize the correct answers to questions while minimizing the answers that go from an initial correct answer to an incorrect answer. Focusing on specific groups of clinicians (eg, those in early years of practice or those with less experience assessing and applying research findings) or in certain situations (eg, constrained time or posing difficult questions outside the domain of the clinician) may also address the potential for automated assistance of PubMed searching. Other research has shown that an interface in PubMed leads to better question answering if the search entry screen required clinicians to enter concepts related to patients or populations, intervention, comparison, and outcome (PICO) aspects of the questions. [49] Comparisons across systems are also warranted, taking into account quality (eg, Google), access (eg, clinicians working inside and outside academic institutions), and cost (eg, UptoDate).

We have shown that complex studies of searching can be done through the Internet. We also have reinforced that clinician searching in PubMed produces an absolute improvement of approximately 10% in clinician ability to correctly answer clinical questions. This 10% improvement is consistent with other similar studies [10,12,17,18] and includes an absolute improvement (incorrect answers to correct answers) of approximately 20% and a decrement of approximately 10% (correct answers to incorrect answers).

## Abbreviations

MORE: McMaster Online Rating of Evidence
PICO: populations, intervention, comparison, and outcome
